# Direct comparison of the effects of intravenous kisspeptin-10, kisspeptin-54 and GnRH on gonadotrophin secretion in healthy men

**DOI:** 10.1093/humrep/dev143

**Published:** 2015-06-18

**Authors:** C.N. Jayasena, A. Abbara, S. Narayanaswamy, A.N. Comninos, R. Ratnasabapathy, P. Bassett, J.T. Mogford, Z. Malik, J. Calley, M.A. Ghatei, S.R. Bloom, W.S. Dhillo

**Affiliations:** 1 Department of Investigative Medicine, Imperial College London, Hammersmith Hospital, 6th Floor, Commonwealth Building, London W12 0NN, UK; 2 Statsconsultancy Ltd, 40 Longwood Lane, Amersham, Bucks HP7 9EN, UK

**Keywords:** kisspeptin-10, kisspeptin-54, GnRH, LH, FSH

## Abstract

**STUDY QUESTION:**

How potently does the novel hypothalamic stimulator of reproduction, kisspeptin, increase gonadotrophin secretion when compared with GnRH in healthy men?

**SUMMARY ANSWER:**

At the doses tested, intravenous administration of either of two major kisspeptin isoforms, kisspeptin-10 and -54, was associated with similar levels of gonadotrophin secretion in healthy men; however, GnRH was more potent when compared with either kisspeptin isoform.

**WHAT IS KNOWN ALREADY:**

Kisspeptin-10 and -54 are naturally occurring hormones in the kisspeptin peptide family which potently stimulates endogenous GnRH secretion from the hypothalamus, so have the potential to treat patients with reproductive disorders. Rodent studies suggest that kisspeptin-54 is more potent when compared with kisspepitn-10; however, their effects have not previously been directly compared in humans, or compared with direct pituitary stimulation of gonadotrophin secretion using GnRH.

**STUDY DESIGN, SIZE AND DURATION:**

A single-blinded placebo controlled physiological study was performed from January to December 2013. Local ethical approval was granted, and five participants were recruited to each dosing group.

**PARTICIPANTS/MATERIALS, SETTING, METHODS:**

Healthy men were administered vehicle, kisspeptin-10, kisspeptin-54 and GnRH intravenously for 3 h on different study days. Each hormone was administered at 0.1, 0.3 and 1.0 nmol/kg/h doses (*n* = 5 subjects per group). Regular blood sampling was conducted throughout the study to measure LH and FSH. Study visits were conducted at least a week apart.

**MAIN RESULTS AND THE ROLE OF CHANCE:**

Serum LH and FSH levels were ∼3-fold higher during GnRH infusion when compared with kisspeptin-10 and ∼2-fold higher when compared with kisspeptin-54 [mean area under the curve serum LH during infusion (in hours times international units per litre, h.IU/l): 10.81 ± 1.73, 1.0 nmol/kg/h kisspeptin-10; 14.43 ± 1.27, 1.0 nmol/kg/h kisspeptin-54; 34.06 ± 5.18, 1.0 nmol/kg/h GnRH, *P* < 0.001 versus kisspeptin-10, *P* < 0.01 versus kisspeptin-54].

**LIMITATIONS, REASONS FOR CAUTION:**

This study had a small sample size.

**WIDER IMPLICATIONS OF THE FINDINGS:**

Kisspeptin offers a novel means of stimulating the reproductive axis. Our data suggest that kisspeptin stimulates gonadotrophin secretion less potently when compared with GnRH; however, kisspeptin may stimulate gonadotrophins in a more physiological manner when compared with current therapies. Kisspeptin is emerging as a future therapeutic agent, so it is important to establish which kisspeptin hormones could be used to treat patients with infertility. Results of this study suggest that either isoform has similar effects on reproductive hormone secretion in healthy men when administered intravenously.

**STUDY FUNDING/COMPETING INTERESTS:**

This work is funded by grants from the MRC and NIHR and is supported by the NIHR Imperial Biomedical Research Centre Funding Scheme. C.N.J. is supported by an NIHR Clinical Lectureship. A.A. is supported by Wellcome Trust Research Training Fellowships. A.N.C. is supported by Wellcome Trust Translational Medicine Training Fellowship. W.S.D. is supported by an NIHR Career Development Fellowship.

## Introduction

Ten per cent of couples are infertile, and this proportion is likely to rise due to the increasing age of couples attempting pregnancy within economically developed countries (NHS Choices, 2014). There is, therefore, an urgent need to develop novel therapeutic avenues for treating patients with infertility. Kisspeptin is a group of recently identified RF-amide peptide hypothalamic hormones which are currently being evaluated as potential novel therapies for infertility (Ohtaki *et al.*, 2001). Kisspeptin hormones are essential for human fertility and act by stimulating GnRH release (Ohtaki *et al.*, 2001). Inactivating mutations in the kisspeptin signalling pathway cause pubertal failure, and activating mutations cause precocious puberty (de Roux *et al.*, 2003; Seminara *et al.*, 2003; Teles *et al.*, 2008; Topaloglu *et al.*, 2012). Exogenous kisspeptin potently stimulates the secretion of the pituitary gonadotrophin hormones (LH and FSH) in numerous mammalian species including rats (Irwig *et al.*, 2004), mice (Gottsch *et al.*, 2004), sheep (Caraty *et al.*, 2007), cows (Kadokawa *et al.*, 2008), monkeys (Shahab *et al.*, 2005) and humans (Dhillo *et al.*, 2005, 2007; Jayasena *et al.*, 2009, 2010; Chan *et al.*, 2011, 2012; George *et al.*, 2011; Young *et al.*, 2013). Kisspeptin has been administered to human subjects without any significant acute or chronic adverse effects.

Furthermore, we have recently demonstrated that exogenous kisspeptin-54 can stimulate egg maturation in women with infertility undergoing *in vitro* fertilization treatment (Jayasena *et al.*, 2014), and Phase 2 studies are currently underway evaluating the effects of the kisspeptin analogue, TAK-448, on reproductive hormone secretion in healthy men (MacLean *et al.*, 2014). Kisspeptin is, therefore, a newly discovered family of hormones which is the subject of intense clinical investigation for its potential role in the treatment of patients with infertility.

Members of the kisspeptin family share a common binding site to the kisspeptin receptor and are denoted by amino acid length (Ohtaki *et al.*, 2001). Unfortunately, early human studies investigating the effect of kisspeptin on the reproductive system have used either kisspeptin-10 or kisspeptin-54 (full-length kisspeptin), but not both isoforms. This is problematic, since clinically important differences are likely to exist between kisspeptin-10 and -54. First, kisspeptin-54 has been observed to stimulate gonadotrophin secretion when administered through the subcutaneous or intravenous routes in all subject groups tested (Dhillo *et al.*, 2005, 2007; Jayasena *et al.*, 2009, 2010). Several studies suggest that Kisspeptin-10 stimulates gonadotrophin secretion when administered intravenously (Chan *et al.*, 2011; George *et al.*, 2011; Jayasena *et al.*, 2011), but one study failed to observe a significant stimulation of gonadotrophin secretion following subcutaneous bolus injection of kisspeptin-10 to healthy women during the follicular phase of menstrual cycle (Jayasena *et al.*, 2011). Differences between the effects of kisspeptin-10 and -54 may be related to our previous observations that the plasma half-live of kisspeptin-10 is ∼6-fold shorter when compared with kisspeptin-54 (Dhillo *et al.*, 2005; Jayasena *et al.*, 2011). In addition, the shorter amino acid sequence of kisspeptin-10 makes it far less expensive to synthesize when compared with kisspeptin-54. Various dosing regimens and human models have been evaluated during previous clinical studies; accordingly, the relative potencies of kisspeptin-10 and -54 stimulating gonadotrophin secretion in humans remain unknown. Furthermore, it is not known how potently kisspeptin-10 and -54 stimulate gonadotrophin secretion in humans when compared with GnRH, which itself may be used to treat patients with infertility. Establishing the potencies of kisspeptin hormones is important for the future development of kisspeptin-based therapies to treat patients with infertility.

We performed a single-blinded placebo controlled study to compare directly for the first time, the effects kisspeptin-10, kisspeptin-54 and GnRH on gonadotrophin secretion in healthy male volunteers, when administered at equimolar doses using a constant 3 h intravenous infusion regimen.

## Materials and Methods

### Ethical approval

Ethical approval was granted by the Local Ethics Research Committee (registration: 12LO/0507). Written informed consent was obtained from all subjects. This study was performed in accordance with the Declaration of Helsinki.

### Subjects

Ten healthy male volunteers were recruited through advertisements in local newspapers. Responders to adverts were evaluated with a detailed medical history, clinical examination, electrocardiogram and blood tests as follows: full blood count, renal profile, liver profile, bone profile, random glucose, thyroid profile, LH, FSH, testosterone and sex hormone-binding globulin. Participants were included within the study if they fulfilled the following criteria: age between 18 and 45 years, absence of significant systemic disease co-morbidity, absence of therapeutic or recreational drug use, normal clinical and biochemical reproductive function, absence of blood donation in the preceding 3 months. Ten subjects participated in the study, with a summary of baseline characteristics provided in Table I.

**Table I:** Baseline characteristics of study volunteers.

Participant no.	Age (years)	Weight (kg)	BMI (kg/m^2^)	LH (IU/l)	FSH (IU/l)	Testosterone (nmol/l)
1	25	75.9	26.5	4.40	2.21	18.78
2	23	82.7	24.1	4.69	2.67	31.40
3	34	73.1	23.9	5.16	4.25	16.05
4	20	68.4	23.6	2.11	1.85	23.97
5	28	88.4	25.7	4.03	3.16	12.29
6	43	66.3	19.8	2.01	2.55	22.91
7	30	68.7	25.2	3.10	2.09	13.65
8	30	62.2	20.2	1.78	1.66	29.75
9	26	71.3	23.4	2.61	2.84	17.05
10	30	83.9	23.7	5.30	1.74	20.52
Mean ± SEM	28.9 ± 2.02	74.10 ± 2.69	23.61 ± 0.68	3.52 ± 0.43	2.50 ± 0.25	20.60 ± 2.03

### Peptides

Gonadorelin 100 µg (GnRH) was purchased from Intrapharm laboratories Ltd (Maidenhead, Berks, UK). It was reconstituted with the sterile solvent supplied containing 2% benzyl alcohol and water for injection. Kisspeptin-10 and kisspeptin-54 were synthesized by Bachem UK (Liverpool, UK). Both kisspeptin isoforms were purified and tested as described previously (Dhillo *et al.*, 2005; Jayasena *et al.*, 2011). Vials of freeze-dried kisspeptin were stored at −20°C and reconstituted in 0.5 ml of 0.9% saline.

### Study protocol

**Figure 1:**
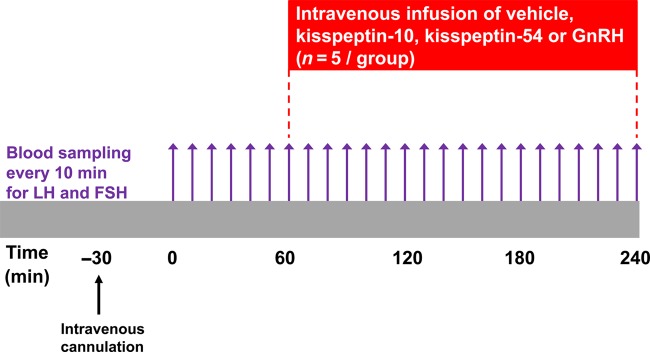
Protocol diagram for study visits in healthy male subjects. Blood samples taken every 10 min from *t* = 0 to *t* = 240 min for serum LH and FSH. Blood samples were also taken for plasma kisspeptin IR at *t* = 0, 60, 70, 80, 90, 120, 180 and 240 min. After 1 h of baseline blood sampling, the infusions of vehicle, kisspeptin-10, kisspeptin-54 or GnRH were commenced. Maintenance infusion doses were 0.1, 0.3 and 1.0 nmol/kg/h of each peptide. Each participant received the same dose of each peptide in random order at least 1 week apart (*n* = 5/group).

Patients were admitted to our clinical investigation unit in the morning and asked to lay supine (see Fig. 1 for protocol summary). Baseline blood sampling was performed at 10 min intervals between 0 and 60 min. A single-blinded 3 h continuous intravenous infusion of gelofusin (vehicle), kisspeptin-10 (0.10, 0.30 or 1.00 nmol/kg/h), kisspeptin-54 (0.10, 0.30 or 1.00 nmol/kg/h) or GnRH (0.10, 0.30 or 1.00 nmol/kg/h) was commenced at time 60 min and continued until 240 min (*n* = 5 subjects per group; see Table II for dose allocation of subjects). The doses selected during this study were based on previous studies that have demonstrated an LH rise during intravenous infusion of kisspeptin-54 in healthy men (Dhillo *et al.*, 2005). A 2-fold higher infusion rate was administered during the first 30 min of each infusion, in order to achieve steady-state plasma levels (Edwards *et al.*, 1999). Therefore, total doses of 0.35, 1.05 or 3.50 nmol/kg peptide were administered during 3 h infusions with maintenance administration rates of 0.10, 0.30 or 1.00 nmol/kg/h, respectively. Blood samples were taken through a cannula at 10 min intervals from *t* = 0 to *t* = 240 min to measure gonadotrophin levels. Blood samples were also taken for plasma kisspeptin immunoreactivity (kisspeptin IR) and at *t* = 0, 60, 70, 80, 90, 120, 180 and 240 min. Study visits for individual patients were scheduled a minimum of a week apart and performed in a random order.

**Table II:** Allocation of healthy men to infusion treatment groups.

Treatment group	Dose	Subject number				
Vehicle	—	2	3	9	10	8
KP10, KP54 or GnRH	0.1 nmol/kg/h	1	4	5	6	7
KP10, KP54 or GnRH	0.3 nmol/kg/h	1	4	7	8	10
KP10, KP54 or GnRH	1.0 nmol/kg/h	2	3	5	6	9

Ten different 3 h intravenous infusions were administered in healthy men: vehicle; 0.1, 0.3 or 1.0 nmol/kg/h kisspeptin-10 (KP10); 0.1, 0.3 or 1.0 nmol/kg/h kisspeptin-54 (KP54); 0.1, 0.3 or 1.0 nmol/kg/h GnRH. In order to directly compare the effects of equimolar doses of kisspeptin-10, kisspeptin-54 and GnRH, the same combination of subjects (numbered 1–10) received the same dose of each peptide. Study visits for individual patients were scheduled a minimum of a week apart and performed in the random order.

### Analytical methods

Serum LH and FSH levels were measured at all time points using automated chemiluminescent immunoassays (Abbott Diagnostics, Maidenhead, UK). Reference ranges for males were as follows: LH 4–14 IU/l; FSH 1.5–8 IU/l. The respective inter-assay coefficients of variation for each assay were: 4.1 and 3.4% (LH) and 4.1 and 3.5% (FSH). Analytical sensitivities were: 0.5 IU/l (LH) and 0.05 IU/l (FSH). Limits of detectability for each assay were: 0.07 IU/l (LH) and 0.05 IU/l (FSH).

Measurement of plasma kisspeptin IR was performed using an established radioimmunoassay (Dhillo *et al.*, 2005, 2007). The antibody used to measure kisspeptin IR has 100% cross-reactivity with kisspeptin-10 and -54 (Dhillo *et al.*, 2005).

### Statistical methods

Cumulative changes in hormone secretion were quantified by calculating area under the curve (AUC) values using Prism (GraphPad Inc., La Jolla, CA, USA); mean AUC values for each peptide and dose were compared using two-way ANOVA with the Bonferroni's *post hoc* analysis test. *P* < 0.05 was considered statistically significant.

## Results

### Levels of circulating kisspeptin during 3 h intravenous infusion of kisspeptin-10, kisspeptin-54 or GnRH

**Figure 2:**
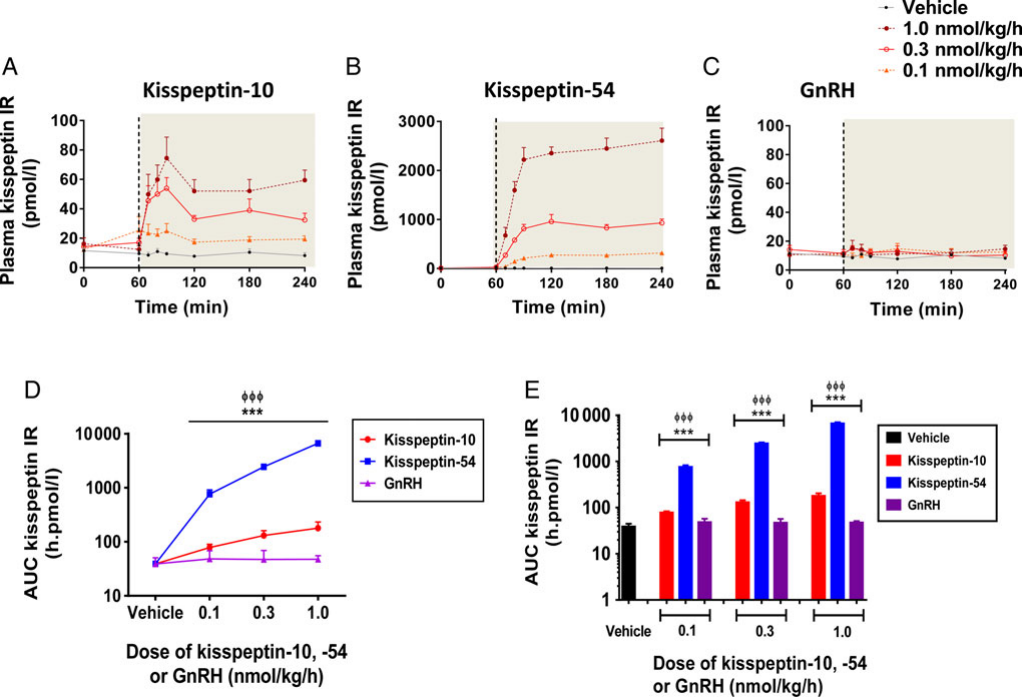
Plasma kisspeptin IR during intravenous administration of kisspeptin-10, kisspeptin-54 and GnRH to healthy men. Time profiles of mean plasma kisspeptin IR are presented during infusion of vehicle, kisspeptin-10 (**A**), kisspeptin-54 (**B**) and GnRH (**C**), at the following doses: 0.1, 0.3 and 1.0 nmol/kg/h. The infusion period commenced at 60 min and is represented by the grey shaded area. Grey filled circles, vehicle; orange filled triangles, 0.1 nmol/kg/h; red clear circles, 0.3 nmol/kg/h; maroon filled circles, 1.0 nmol/kg/h. Mean AUC plasma kisspeptin IR is presented for each infusion group: graph **D** shows data as linear curves and graph **E** as bar graphs. Vehicle in black, kisspeptin-10 in red, kisspeptin-54 in blue and GnRH in purple. Data are presented as the mean ± SEM. *N* = 5/group. ***P < 0.001 for GnRH versus kisspeptin-54. ^ΦΦΦ^*P* < 0.001 for GnRH versus kisspeptin-10.

Time profiles for plasma kisspeptin IR are presented for vehicle and each dose of kisspeptin-10, kisspeptin-54 and GnRH in healthy men (Fig. 2A–C), and cumulative levels of kisspeptin IR (AUC) are also presented (Fig. 2D and E). As expected, levels of plasma kisspeptin IR were not elevated significantly during intravenous infusion of vehicle or any tested dose (0.1, 0.3 and 1.0 nmol/kg/h) of GnRH in healthy men (Fig. 2A–E). Dose-dependent elevations in plasma kisspeptin IR were observed during infusion kisspeptin-54 and to a lesser extent during kisspeptin-10 infusion. At the highest dose (1.0 nmol/kg/h), levels of AUC kisspeptin IR were 37-fold higher during kisspeptin-54 infusion when compared with kisspeptin-10 and 170-fold higher when compared with vehicle (mean AUC plasma kisspeptin IR during infusion in h pmol/l: 179 ± 24, 1.0 nmol/kg kisspeptin-10; 6650 ± 397, 1.0 nmol/kg kisspeptin-54, *P* < 0.001 versus vehicle, *P* < 0.001 versus 1.0 nmol/kg kisspeptin-10).

### Levels of serum LH during 3 h intravenous infusion of kisspeptin-10, kisspeptin-54 or GnRH

**Figure 3:**
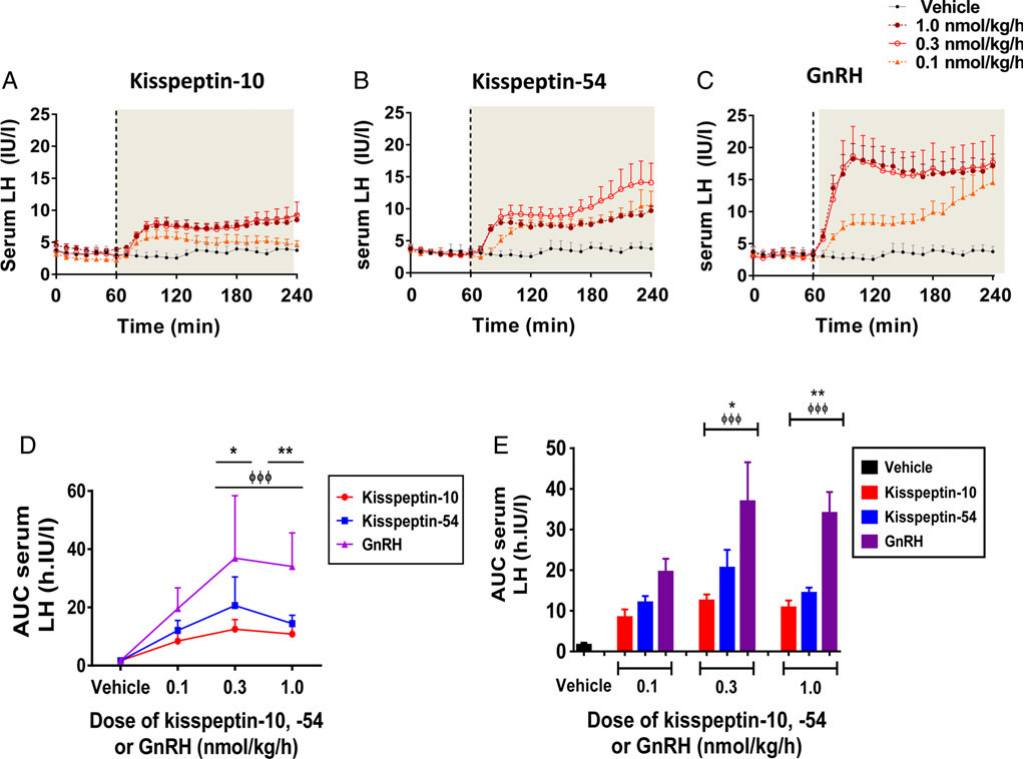
Change in serum LH levels during intravenous administration of kisspeptin-10, kisspeptin-54 and GnRH to healthy men. Time profiles of mean change in serum LH are presented during infusion of vehicle, kisspeptin-10 (**A**), kisspeptin-54 (**B**) and GnRH (**C**), at the following doses: 0.1, 0.3 and 1.0 nmol/kg/h. The infusion period commenced at 60 min and is represented by the grey shaded area. Grey filled circles, vehicle; orange filled triangles, 0.1 nmol/kg/h; red clear circles, 0.3 nmol/kg/h; maroon filled circles, 1.0 nmol/kg/h. Mean change in AUC serum LH is presented for each infusion group: graph **D** shows data as linear curves and graph **E** as bar graphs. Vehicle in black; kisspeptin-10 in red; kisspeptin-54 in blue; GnRH in purple. Data are presented as the mean ± SEM. *N* = 5/group. *P < 0.05 for GnRH versus KP54. **P < 0.01 for GnRH versus kisspeptin-54. ^ΦΦΦ^*P* < 0.001 for GnRH versus kisspeptin-10.

Time profiles for serum LH are presented for vehicle and each dose of kisspeptin-10, kisspeptin-54 and GnRH in healthy men (Fig. 3A–C), and cumulative levels of AUC serum LH are also presented (Fig. 3Dand E). As expected, levels of serum LH were not elevated significantly during intravenous infusion of vehicle in healthy men (Fig. 3A–E). Dose-dependent elevations in serum LH were observed during infusion of GnRH and to a lesser extent during kisspeptin-10 and kisspeptin-54 infusions. For all three hormones, peak levels of LH secretion were observed at the 0.3 nmol/kg/h dose; at this dose, levels of AUC serum LH were 3-fold higher during GnRH infusion when compared with kisspeptin-10 and 2-fold higher when compared with kisspeptin-54 (mean AUC serum LH during infusion in h IU/l: 10.81 ± 1.73, 1.0 nmol/kg/h kisspeptin-10; 14.43 ± 1.27, 1.0 nmol/kg/h kisspeptin-54; 34.06 ± 5.18, 1.0 nmol/kg/h GnRH, *P* < 0.001 versus kisspeptin-10, *P* < 0.01 versus kisspeptin-54).

### Levels of serum FSH during 3 h intravenous infusion of kisspeptin-10, kisspeptin-54 and GnRH

**Figure 4:**
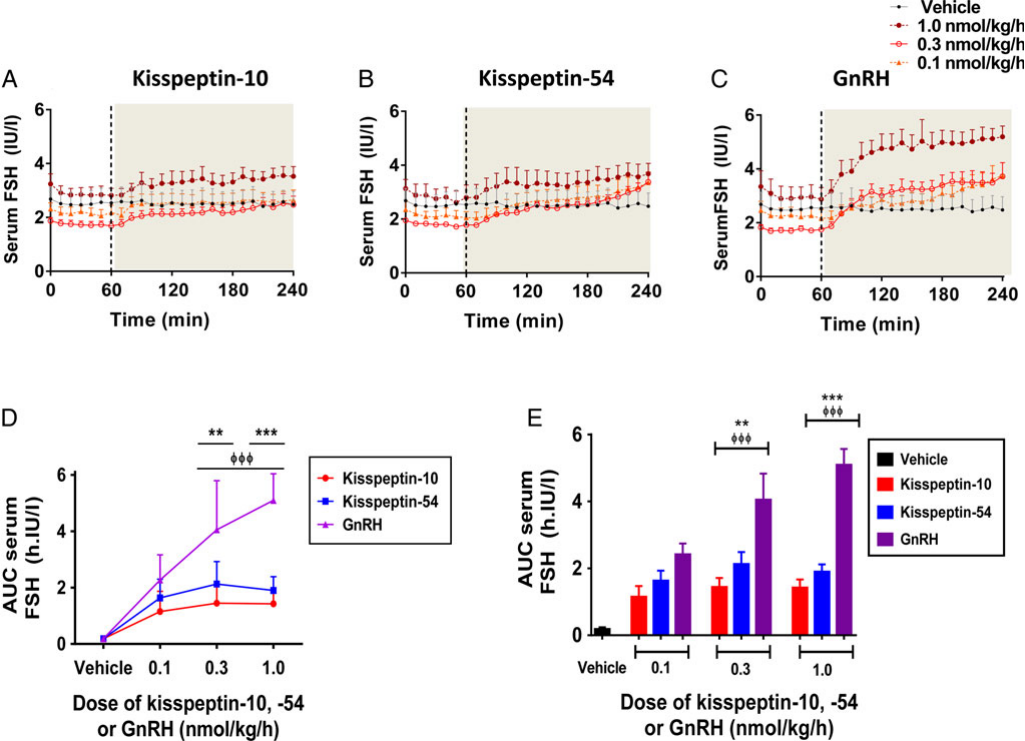
Change in serum FSH levels during intravenous administration of kisspeptin-10, kisspeptin-54 and GnRH to healthy men. Time profiles of mean change in serum FSH are presented during infusion of vehicle, kisspeptin-10 (**A**), kisspeptin-54 (**B**) and GnRH (**C**), at the following doses: 0.1, 0.3 and 1.0 nmol/kg/h. The infusion period commenced at 60 min and is represented by the grey shaded area. Grey filled circles, vehicle; orange filled triangles, 0.1 nmol/kg/h; red clear circles, 0.3 nmol/kg/h; maroon filled circles, 1.0 nmol/kg/h. Mean change in AUC serum FSH is presented for each infusion group: graph **D** shows data as linear curves and graph **E** as bar graphs. Vehicle in black; kisspeptin-10 in red; kisspeptin-54 in blue; GnRH in purple. Data are presented as the mean ± SEM. *N* = 5/group. **P < 0.01 for GnRH versus kisspeptin-54. ***P < 0.001 for GnRH versus kisspeptin-54. ^ΦΦΦ^*P* < 0.001 for GnRH versus kisspeptin-10.

Time profiles for serum FSH are presented for vehicle and each dose of kisspeptin-10, kisspeptin-54 and GnRH in healthy men (Fig. 4A–C), and cumulative levels of AUC serum LH are also presented (Fig. 4D and E). As expected, levels of serum FSH were not elevated significantly during intravenous infusion of vehicle in healthy men (Fig. 4A–E). Dose-dependent elevations in serum FSH were observed during infusion of GnRH and to a lesser extent during kisspeptin-10 and kisspeptin-54 infusions. Peak levels of FSH secretion during GnRH were observed at the 1.0 nmol/kg/h dose; at this dose, levels of AUC serum FSH were over 3-fold higher during GnRH infusion when compared with kisspeptin-10 and over 2-fold higher when compared with kisspeptin-54 (mean AUC increase in serum FSH during infusion in h IU/l: 1.43 ± 0.24, 1.0 nmol/kg/h kisspeptin-10; 1.90 ± 0.22, 1.0 nmol/kg/h kisspeptin-54, *P* < 0.01 versus vehicle; 4.69 ± 0.55, 1.0 nmol/kg/h GnRH, *P* < 0.001 versus 1.0 nmol/kg/h kisspeptin-10, *P* < 0.001 versus 1.0 nmol/kg/h kisspeptin-54).

## Discussion

Direct stimulation of the hypothalamus using kisspeptin administration offers an attractive novel potential avenue for treating patients with infertility, since it causes the release of an endogenous pool of GnRH, which in turn stimulates physiological levels of gonadotrophin secretion (Gottsch *et al.*, 2004; Messager *et al.*, 2005; Shahab *et al.*, 2005). Kisspeptin, therefore, stimulates the reproductive axis in a self-limiting manner, which is more physiological when compared with direct pituitary or gonadal stimulation with GnRH analogues or gonadotrophin preparations, respectively. Studying the clinical effects of kisspeptin is complicated by the presence of two major studied isoforms of kisspeptin -10 and -54, which have differential pharmacokinetic profiles, potential routes of administration and manufacturing costs (Dhillo *et al.*, 2005, 2007; Chan *et al.*, 2011; George *et al.*, 2011; Jayasena *et al.*, 2011). Furthermore, the effects of neither kisspeptin hormone on gonadotrophin secretion have been directly compared with the effects of GnRH. Our data suggest for the first time that at the doses tested, GnRH stimulates gonadotrophins more potently when compared with either kisspeptin hormone in healthy men, but that kisspeptin-10 and -54 have broadly similar potencies of action. These data have important implications for the development of future therapies for infertility based upon kisspeptin.

The effects of the long and short forms of kisspeptin have been compared directly in rodents. Intravenous bolus injection of 3.0 nmol/kg kisspeptin-52 (the rodent homologue of kisspeptin-54) stimulated ∼30% more LH secretion in male rats when compared with an equimolar dose of kisspeptin-10 (Tovar *et al.*, 2006). Furthermore, subcutaneous bolus injection of 1 or 50 nmol kisspeptin-54 stimulated ∼6-fold more LH secretion in male rats when compared with an equimolar dose of kisspeptin-10 (Thompson *et al.*, 2006). It is interesting to note that kisspeptin-10 and -54 infusions were associated with comparable gonadotrophin responses despite levels of plasma kisspeptin IR being significantly higher during the kisspeptin-54 infusion. The likely explanation for this finding is the much shorter *in vitro* half-life of kisspeptin-10 of 55 s (Seminara *et al.*, 2003) and *in vivo* plasma half-life of kisspeptin-10 of 4 min (Jayasena *et al.*, 2011) compared with kisspeptin-54 of 27.6 min (Dhillo *et al.*, 2005) which would lead to rapid degradation of kisspeptin-10 in comparison with kisspeptin-54. Hence, this difference in half-life between kisspeptin-10 and -54 would be expected to result in lower steady-state levels of plasma kisspeptin IR during kisspeptin-10 infusion compared with kisspeptin-54 infusion as observed in this study.

Kisspeptin-54 has consistently been observed to stimulate gonadotrophin secretion when administered by subcutaneous bolus injection in healthy women in each phase of the menstrual cycle, in addition to women with hypothalamic amenorrhoea (Dhillo *et al.*, 2007; Jayasena *et al.*, 2009). In contrast, subcutaneous bolus injection of kisspeptin-10 appears not to stimulate gonadotrophin secretion significantly in women during the follicular phase of the menstrual cycle (Jayasena *et al.*, 2011). However, we cannot exclude the possibility that subcutaneous bolus injection of kisspeptin-10 in other human models (e.g. healthy women during the pre-ovulatory phase of the menstrual cycle and women with hypothalamic amenorrhoea) could result in gonadotrophin release.

GnRH has been compared with kisspeptin in only a few animal studies. Tovar *et al.* (2006) found that male rats had a greater LH response to i.v. GnRH compared with equimolar doses of IV kisspeptin-10, when it was given 120 min after the last pulse of kisspeptin-10 (given every 75 min for the preceding 450 min). Thompson *et al.* (2009) showed that a single 50 nmol bolus of s.c. kisspeptin-54 resulted in similar gonadotrophin release when compared with equimolar doses of GnRH in rats. In adult female goats in the luteal phase, kisspeptin-10 or GnRH caused a rise in gonadotrophins; however, GnRH produced a more gradual rise that achieved greater peak levels of gonadotrophins compared with kisspeptin-10 when given at the same dose (Hashizume *et al.*, 2010). Male juvenile rhesus monkeys primed with intermittent IV GnRH showed that on receiving an IV bolus of kisspeptin-10, their LH pulses were of similar magnitude to that seen during the preceding GnRH priming; however, the dose of kisspeptin was almost 10 fold higher than that of GnRH (Plant *et al.*, 2006).

There are a number of possible reasons why the maximal effects of kisspeptin-10 or -54 (at 0.3 nmol/kg/h) on LH secretion were significantly lower than the maximal effects of GnRH (at 1.0 nmol/kg/h) in healthy men. The likely predominant reason is that endogenous GnRH released by kisspeptin may stimulate a submaximal level of gonadotrophin secretion from the pituitary. Second, it is possible that not all kisspeptin can penetrate the hypothalamus from the circulation. Kisspeptin has been shown to activate the release of GnRH from nerve terminals at the median eminence, which lacks a complete blood brain barrier (bbb) (d'Anglemont de Tassigny et al., 2010). However, it is not known whether additional saturable transport mechanisms for kisspeptin exist across the bbb. Finally, the majority but not all GnRH neurons express the kisspeptin receptor (∼77% in adult male rats), which implies that a subset of GnRH neurons lack the receptor to respond to kisspeptin (Irwig *et al.*, 2004). These factors clearly contribute to the reduced potency of kisspeptin to stimulate reproductive function when compared with GnRH; however, one could speculate whether these same factors also confer a therapeutic advantage over exogenous GnRH, by stimulating a level of gonadotrophin secretion which does not exceed physiological levels. Future studies are needed to determine if such an action could prevent adverse consequences of excessive gonadotrophin stimulation, such as the ovarian hyperstimulation syndrome during *in vitro* fertilization therapy (Elchalal and Schenker, 1997).

It is important to consider the strengths and weakness of the study. Each kisspeptin isoform has relative advantages over the other isoform. For instance, kisspeptin-54 but not kisspeptin-10 can be administered subcutaneously due to its long half-life; however, kisspeptin-10 is substantially cheaper to manufacture when compared with kisspeptin-54, due to its short amino acid sequence. This study provides the first direct and objective comparison of kisspeptin-10 and -54 administration in human subjects. We recognize that this was a small study primarily aimed to compare the potencies of two novel kisspeptin hormones with an established stimulator of LH secretion, GnRH. However, ethical considerations prohibited us from giving vehicle plus each of the three doses of each three tested hormones to individual subjects, which may have accounted for some of the observed variation in data. It would be interesting to extend the duration of blood sampling following kisspeptin and GnRH infusions beyond 4 h, in order to accurately compare the levels of sex steroid secretion in subjects.

In summary, we have directly compared for the first time, the effects of kisspeptin-10, kisspeptin-54 and GnRH on gonadotrophin secretion in healthy men. At the doses tested, intravenous administration of kisspeptin-10 and -54 was associated with similar levels of gonadotrophin secretion in healthy men, but GnRH was more potent when compared with either kisspeptin isoform. These data provide important information for the future development of kisspeptin-based therapies to treat patients with infertility.

## Authors' roles

C.J. and W.S.D. conceived the study. C.J., W.S.D. and A.A. wrote the first draft of the manuscript. A.A., S.N., A.C., R.R., J.M. and J.C. conducted the studies and collected the samples. A.A., S.N., A.C., J.M., Z.M., J.C., M.A.G. and S.R.B. carried out the laboratory analysis. A.A., C.J., S.N. and Z.M. performed the data analysis and drafted the results and figures. P.B. conducted detailed statistical analysis. All authors reviewed and edited the manuscript and approved the final version of the manuscript.

## Funding

This work is funded by grants from the MRC and NIHR. C.N.J. is supported by an NIHR Clinical Lectureship. A.A. is supported by Wellcome Trust Research Training Fellowships. A.N.C. is supported by Wellcome Trust Translational Medicine Training Fellowship. W.S.D. is supported by an NIHR Career Development Fellowship. Funding to pay the Open Access publication charges for this article was provided by the Wellcome Trust.

## Conflict of interest

None declared.
